# In Vitro Lung Models and Their Application to Study SARS-CoV-2 Pathogenesis and Disease

**DOI:** 10.3390/v13050792

**Published:** 2021-04-28

**Authors:** Natalie Heinen, Mara Klöhn, Eike Steinmann, Stephanie Pfaender

**Affiliations:** Department of Molecular and Medical Virology, Ruhr-University Bochum, 44801 Bochum, Germany; Natalie.Heinen@rub.de (N.H.); Mara.Kloehn@rub.de (M.K.); Eike.Steinmann@rub.de (E.S.)

**Keywords:** SARS-CoV-2, in vitro lung model, cell culture, human airway epithelial cell culture, human lung organoids, lung-on-a-chip, ex vivo lung, air–liquid interface

## Abstract

SARS-CoV-2 has spread across the globe with an astonishing velocity and lethality that has put scientist and pharmaceutical companies worldwide on the spot to develop novel treatment options and reliable vaccination for billions of people. To combat its associated disease COVID-19 and potentially newly emerging coronaviruses, numerous pre-clinical cell culture techniques have progressively been used, which allow the study of SARS-CoV-2 pathogenesis, basic replication mechanisms, and drug efficiency in the most authentic context. Hence, this review was designed to summarize and discuss currently used in vitro and ex vivo cell culture systems and will illustrate how these systems will help us to face the challenges imposed by the current SARS-CoV-2 pandemic.

## 1. Introduction

### 1.1. Anatomy and Cellular Characteristics of the Human Respiratory System

**Figure 1:**
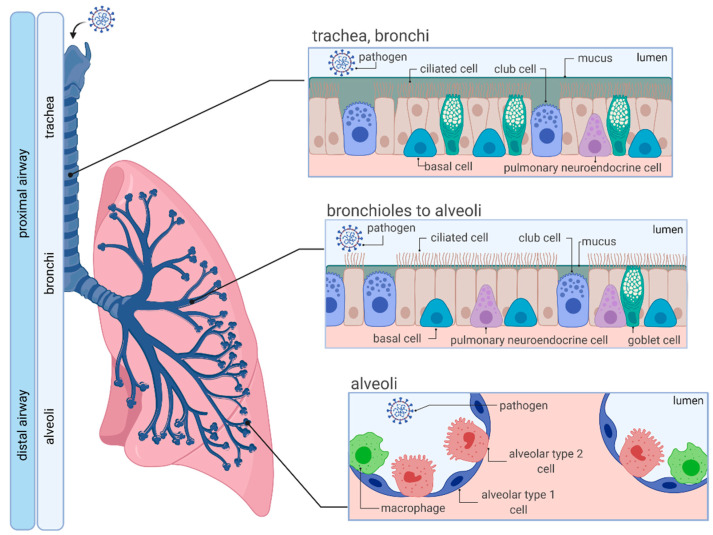
Organization and cellular characteristics of the human respiratory airway. Adapted from “Respiratory Epithelium”, by BioRender.com (accessed on 22 April 2021) (2021). Retrieved from https://app.biorender.com/biorender-templates (accessed on 21 April 2021).

The respiratory system is a complex network that is formed by the upper (nasal cavity, pharynx and larynx), and the lower respiratory tract, which is further divided into the proximal (trachea, bronchi and bronchioles) and distal airway (respiratory bronchioles and alveoli) (Figure 1). The entire human respiratory tract, which is lined with polarized airway epithelium (apical side facing air/lumen, basolateral side facing the internal milieu), is structurally diverse and fulfills multiple functions, depending on anatomical location. While the proximal conducting airways function as a gas transport system, alveoli of the distal airway facilitate air exchange and respiration [1].

Structurally, the pseudostratified tracheobronchial epithelium consists of specialized cells including columnar club, ciliated, goblet and basal cells, incorporated into a basement membrane, which facilitate mucus production, mucociliary clearance, mucus secretion, and serve as progenitors for columnar cells [2]. Moving further down the respiratory tree, thinner cuboidal epithelium appears with an increased number of club cells. In contrast, goblet cells become increasingly less frequent until they can no longer be found in the alveoli. The distal alveolar region is lined with two types of epithelial cells (alveolar epithelial type 1 (AT1) and alveolar epithelial type 2 (AT2)). Squamous AT1 cells provide a specialized surface for gas exchange, whereas cuboidal AT2 cells secrete pulmonary surfactant to prevent alveolar collapse during expiration [1,3].

In addition to gas exchange, the human airway epithelium has been recognized to function as the main entry point for several pathogens. Notably, human airway epithelial cells represent a primary target of coronaviruses, including the highly transmissible and pathogenic Severe Acute Respiratory Syndrome Coronavirus 2 (SARS-CoV-2) [4,5,6]. To defend itself from pathogens such as SARS-CoV-2, epithelial cells have adapted numerous strategies that include tightly packed epithelial cells forming tight junctions, mucosal epithelium trapping invading pathogens in the secreted mucus barrier and the expression of pattern-recognition receptors. Furthermore, within the so-called mucociliary escalator, invaded pathogens are cleared by coordinated cilia beating [7]. Additionally, cells of the immune system (e.g., macrophages) are present to eliminate the intruding pathogen [8,9].

Given the complexity and diversity of the human respiratory system, it is of utmost importance to develop authentic in vitro and ex vivo culture models, which faithfully model basic human pathology and the complexity and diversity of human respiratory tissues to study emerging viral respiratory pathogens, such as SARS-CoV-2.

### 1.2. SARS-CoV-2

Respiratory coronaviruses (CoVs) were first discovered in the 1960s [10] and are commonly found in many vertebrates and humans, where they are known to cause various types of disease. CoVs are a large group of viruses belonging to the family *Coronaviridae*, which are further divided by phylogenetic clustering into 4 genera: *Alphacoronavirus* (αCoV), *Betacoronavirus* (βCoV), *Gammacoronavirus* (γCoV) and *Deltacoronavirus* (δCoV) [11].

So far, four endemic coronaviruses have been identified circulating in the human population, including HCoV-229E (αCoV), HCoV-OC43 (βCoV), HCoV-NL63 (αCoV) and HCoV-HKU1 (βCoV) that are usually causing mild, self-limiting respiratory infections also known as the “common cold” in immunocompetent patients and occasionally respiratory tract infections in immunosuppressed individuals [12]. In contrast, several βCoVs with increasing pathogenicity have emerged during the past two decades. The Severe Acute Respiratory Syndrome CoV (SARS-CoV) outbreak in the Guangdong Province of China in 2002–2003 lead to over 8400 confirmed cases with a mortality rate of about 11% [13]. In addition, Middle East Respiratory Syndrome CoV (MERS-CoV) emerged on the Arabian Peninsula 10 years later, in 2012, causing a serious series of highly pathogenic respiratory tract infections, leading to over 2800 verified cases with a mortality rate of 35% [12,14].

Most recently, SARS-CoV-2, a betacoronavirus with 96.2% sequence similarity to the bat CoV RaTG13 and 79% sequence similarity to SARS-CoV, emerged, leading to a fast-spreading global pandemic [15,16]. As of 12 March 2021, over 119 million confirmed cases and nearly 2.6 million deaths were reported globally by the World Health Organization [17], demonstrating its remarkably high transmission potential and mortality.

The clinical course of SARS-CoV-2 infection varies significantly from patient to patient in its severity and disease outcome. While some patients experience mild symptoms (such as fever, cough, shortness of breath, sore throat and muscle ache) or are asymptomatic, in others it causes acute respiratory distress syndrome (ARDS), severe sepsis and even death [18,19]. Notably, in some individuals, severe disease progression has been linked to the so-called “cytokine storms”, initiated through rapid virus propagation and uncontrolled inflammation [20].

SARS-CoV-2 is an enveloped, single-stranded positive-sense RNA virus which encodes a 29.9 kb genome. Structurally, virion particles are assembled from four proteins: The spike (S), small envelope (E), membrane (M) and nucleocapsid (N) glycoproteins are involved in host cell receptor recognition, virion assembly and egress, shaping the virion and viral RNA encapsulation, respectively. In addition, 16 non-structural proteins and a variety of accessory proteins have been identified to be involved in RNA processing, RNA replication, and survival in the host cell [21].

The main route of transmission is believed to be through respiratory droplets that are released into the air through breathing, coughing, sneezing or talking, a high risk especially upon close contact [22]. SARS-CoV-2 then enters the human respiratory system and enters susceptible cells by binding of the surface spike (S) glycoprotein to its receptor, human angiotensin-converting enzyme 2 (ACE2), expressed on membranes of epithelial cells in the upper and lower airways with very high affinity [23]. Subsequent proteolytic priming of the viral spike protein through cellular proteases (e.g., TMPRSS2) facilitates fusion of the viral envelope with the cell membrane and release of the viral genome into the cells [24]. Afterwards, the virus replicates and spreads within the airways and alveoli.

Within the past year, several vaccines targeting SARS-CoV-2 were developed with about 82 candidates in clinical trials and 182 candidates currently in preclinical development, as of 17 March 2021 [25]. Notably, the first vaccine authorization (mRNA vaccine BNT162B (Pfizer-BioNTech) that encodes for the full-length SARS-CoV-2 spike protein) was issued in December 2020 after successful completion of the third clinical phase [26], and was followed by approval of numerous other vaccines (e.g., AZD1222 [27], mRNA-1273 [28]). There are no specific antiviral molecules approved for the treatment of SARS-CoV-2, up until now, with the exception of the drug remdesivir, which has an emergency use authorization, however, it is no longer recommended by the WHO [29]. This highlights the urgency of identifying and developing novel drug candidates, for which authentic cell culture models are of utmost importance.

Furthermore, the fact that CoVs are classified as zoonotic pathogens that can easily be transmitted from animals to humans implicates the possibility of future outbreaks, which necessitates future extensive studies of coronaviruses.

Recent studies have successfully utilized animal models, including non-human primates, human ACE2 transgenic mice and golden Syrian hamsters, to study SARS-CoV-2 infection [30,31,32] (an in-depth literature review on animal models used for SARS-CoV-2 infection and COVID-19 can be found here [33]).

Ever since the beginning of the COVID-19 pandemic, numerous human-derived in vitro and ex vivo models have been proposed for the study of SARS-CoV-2 pathogenesis and drug development. Thus, this review aims to summarize and compare several available in vitro and ex vivo models used to study human coronavirus infection and disease with emphasis on SARS-CoV-2, critically considering their benefits and limitations.

## 2. In Vitro Cell Culture Models for SARS-CoV-2 Infections

### 2.1. Conventional Cell Lines—Invaluable Tools for Life Sciences

Conventional cell lines are often the first choice when studying viral infections, including respiratory virus infections. Because of their infinite accessibility and high reproducibility, they are especially suitable for high-throughput approaches, and the mechanistic and functional analysis of one-to-one interactions between cell and pathogen [34,35].

In general, the most frequent cell lines utilized for SARS-CoV-2 studies include Vero E6, an immortalized African green monkey kidney cell line [36,37], human adenocarcinoma epithelial cell lines Calu-3 [36] and A549 [37,38], colon carcinoma cell line Caco-2 [36], human embryonic kidney HEK-293T [39] and hepatocellular carcinoma Huh-7 cells [37] (Table 1). Among these cell lines, SARS-CoV-2 replicates most robustly in Calu-3, Caco-2 and Vero E6 cells, followed by moderate replication in Huh-7 cells. In contrast, HEK-293T and A549 cells are incompatible with SARS-CoV-2 infection due to low ACE2 expression [40,41,42], which is frequently bypassed by transduction with lentivirus- or adenovirus (AdV)-based vectors expressing ACE2 [43].

Conventional cell lines can serve as invaluable large-scale screening platforms for antiviral compounds. Riva et al. screened large compound libraries comprising 12,000 small molecules of the Food and Drug Administration (FDA) for SARS-CoV-2 antivirals in Vero E6 cells and reported the identification of 100 molecules that inhibited viral replication of SARS-CoV-2, including 21 drugs that exhibit dose–response relationships [44]. Similarly, Touret et al. utilized Vero E6 and Caco-2 cells to screen potential SARS-CoV-2 replication inhibitors among 1520 FDA-approved compounds. From this study, 90 compounds were identified exhibiting antiviral activity [45]. Additionally, SARS-CoV-2 inhibition was shown for numerous antiviral compounds, including remdesivir, chloroquine and hydroxychloroquine in Vero E6 [46,47] and Calu-3 cells [48].

Finally, conventional cell lines are utilized for the production of SARS-CoV-2 viral particles and diagnostic applications. Vero E6 cells are commonly used as factories to generate high-titer viral particles and to facilitate high-throughput virus production. Detecting neutralizing antibodies in patient sera can be rapidly performed and quantified in conventional cell culture. For instance, our team and others have developed a simple, yet highly relevant, diagnostic system to analyze neutralizing antibodies in a biosafety level 1 environment using a VSV- or HIV-1-based pseudotype system in Vero E6 cells [49,50].

Even though conventional cell lines are easy to handle and enable to simply study the basics of viral infections, these models do not reflect the cellular composition, and lack matrix complexity, tissue diversity and three-dimensional architecture compared to the native lung tissues. Additionally, important aspects of viral tropism, virus–host interactions, disease pathogenesis, transmission and antiviral drug efficacy observed in these cell culture models allow no direct conclusions to be drawn regarding applications in humans. Therefore, results obtained from conventional cell-based studies should be interpreted with caution to avoid drawing premature conclusions for humans. Preferably, in vitro cell culture studies are preferentially complemented with suitable human-derived models. Hence, we will discuss in vitro and ex vivo culture techniques reproducing tissue diversity on a cellular level in the following sections.

### 2.2. Primary Human Airway Epithelial Cell (hAEC) Air–Liquid Interface (ALI) Cultures—The Gold Standard

Human airway epithelial cell (hAEC) cultures are organotypic cell cultures that have been used to study wound repair and cell regeneration [51,52], and have been applied for disease modeling of chronic obstructive pulmonary disease (COPD) [53], cystic fibrosis (CF) [54] and respiratory infectious diseases [6,55,56,57,58,59,60,61,62,63]. While effectively maintaining the specific functionality, architecture and cellular complexity of the human airway, hAEC cultures are capable of authentically reproducing different parts of the human respiratory system [64]. This includes mucus secretion and cilia movement, the protective machinery of the native human lung.

**Figure 2:**
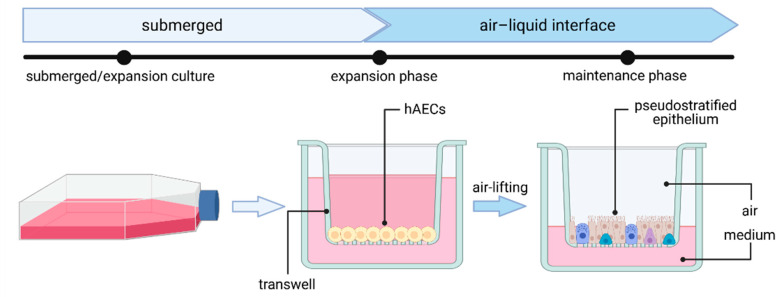
Generation of human airway epithelial cell culture in air–liquid interface (ALI) from human airway epithelial cells (hAECs). Figure created with BioRender.com (accessed on 28 April 2021).

As they serve as the main entry point for several pathogens and as a pathogen defense barrier, human airway epithelial cultures have proven to be invaluable when it comes to studying respiratory viral infections. In general, human airway epithelial cells—including nasal, tracheal, bronchial or alveolar epithelial cells—can be cultured as primary cells isolated from nasal brushes and biopsies or obtained from commercially available cryopreserved cells. Airway epithelial cells are cultured on an air–liquid interface (ALI), where the basolateral and apical site of cells will be exposed to cell culture medium or air, respectively (Figure 2) [64]. First, cells are expanded on a porous membrane insert (transwell). Once cells reach confluency (after 2–4 days), differentiation is induced by airlifting (removing liquid medium). After complete differentiation, many cell types, including ciliated cells, club cells, goblet cells and basal cells, have emerged and form a pseudostratified mucociliary epithelial layer, authentically mimicking the native tissue-like cell polarization found in the human airway.

Previously, hAEC-ALI culture systems have been used to study different coronaviruses [65] and are suitable to study SARS-CoV-2 cell tropism and morphogenesis. For instance, recent studies have reported highest expression of the SARS-CoV-2 receptor ACE2 in nasal cells [66]. Furthermore, infection of hAECs with SARS-CoV-2 revealed that the virus primarily targets ciliated cells and a small percentage of goblet cells, whereas basal cells and club cells were not infected [55,57,67], which is in agreement with a study that reported highest expression levels of ACE2 in ciliated and goblet cells [66]. In contrast, one study found evidence for SARS-CoV-2 infection in basal cells to at least a small extent [68]. Other studies using hAEC-ALI cell culture systems found that viral particles are mainly released to the apical site of HAEs. However, once the epithelial layer is damaged by cytopathic effects (CPE), viral particles can also be released basolaterally [57,67,69]. Additionally, reduction of the epithelial layer integrity, measured by a decrease of transepithelial electrical resistance (TEER), as well as disrupted tight junctions, cilium disorder and shrinking, were reported in SARS-CoV-2-infected cells [57,66,67].

hAEC-ALI cultures have also been used to evaluate the relationship between SARS-CoV-2 and immune responses. Fiege et al. detected induction of ISGs and found a strong positive correlation between levels of SARS-CoV-2 replication and induction of IFN. The same group also observed preferential induction of the ISG MT1F within ciliated cells and significantly lower expression levels of the ISGs DDIT, IFITM3, LY6E, TNFSF10 in cells with the highest levels of SARS-CoV-2 RNA, suggestive of an impaired cellular antiviral immune response [70].

Furthermore, hAEC-ALI cultures have been recognized as a suitable model system to investigate putative drug candidates against coronavirus infection. Recent studies on SARS-CoV [69], MERS-CoV [69] and SARS-CoV-2 [69,71] have elucidated the inhibitory and antiviral effects of numerous compounds, including β-d-N^4^-hydroxycytidine [69], remdesivir [48] and many more [71,72], in hAEC-ALI cultures.

As primary cells are required for the establishment of AECs, limited accessibility and high donor-to-donor variability need to be considered. Nonetheless, hAEC-ALI cultures provide greater physiological relevance than conventional cell culture models. They can efficiently be infected with numerous coronaviruses, including SARS-CoV-2, and allow easy access to the apical surface area. Other than conventional cell lines, hAEC-ALI cell cultures enable the examination of crucial epithelial functions, including cilia beating, ion channel activity, airway surface liquid volume maintenance and mucus secretion. By obtaining hAECs from different individuals, cultivation in ALI can also be used to mimic the inhomogeneous and vast-ranging SARS-CoV-2 virus responses in the human population. Finally, hAEC-ALI cultures serve as a sensitive platform for drug screening and validation, greatly facilitating drug development for SARS-CoV-2-infected patients.

To conclude, establishing human airway ALI cultures may be labor-intensive, but can serve as an indispensable preclinical tool for analyzing human respiratory pathogens, most notably SARS-CoV-2.

### 2.3. Lung Organoids—Innovative Technology

**Figure 3:**
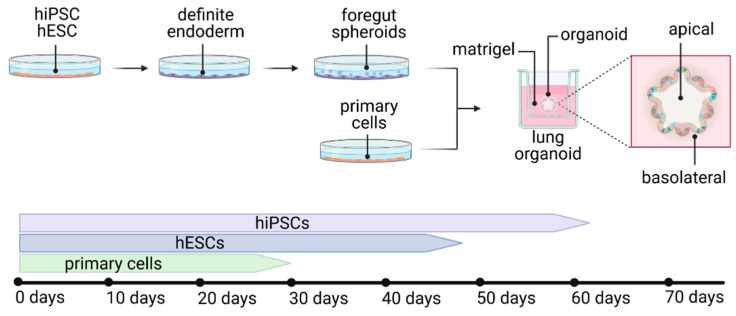
Development of different types of lung organoids from hiPSCs, hESCs or primary cells, with the average duration of development. Figure created with BioRender.com (accessed on 26 March 2021).

Organoids are three-dimensional tissue cultures, recapitulating native organs to a large extent. These tissues develop by self-organization of different types of stem cells, mimicking organ development. Organoids are potentially able to overcome limitations of conventional cell culture or animal models because of the high comparability to the human organ, thus providing a reliable in vitro platform to study viral disease mechanisms and pathogenesis for the development of drug candidates and for applications in personalized medicine. Until now, a variety of organoid protocols for various organs have been developed, including human lung organoids. Lung organoids are derived from human induced pluripotent stem cells (hiPSCs), human embryonic stem cells (hESCs) or primary cells, recapitulating multicellular features [73,74,75,76,77] or alveolar type I and II (AT1 and AT2) cells [77,78,79,80,81,82] (Figure 3). Of note, due to the fact that lung organoids do not recapitulate apical air polarization, the infection and spreading of a virus does differ from its in vivo behavior.

So far, only very limited protocols represent the multicellular composition of a complete functional adult lung [73,75]. Leibel et al. described 2D differentiation of stem cells to definitive endoderm, followed by anterior foregut endoderm and lung progenitor differentiation. Subsequent culturing of cells embedded in a human extracellular matrix (Matrigel) on transwell inserts and supply of defined growth factors and small molecules led to the characteristic development of lung branching and maturation (3D differentiation). In this three-dimensional culture, the organoids developed epithelial and mesenchymal cells from the proximal and distal lung in 35 days. However, this protocol has not yet been used to study SARS-CoV-2, even though the authors mention the suitability of the model for studying respiratory viruses [73]. Similarly, protocols of Spence and his colleagues describe the development of a multicellular, but rather fetal, lung tissue in a developmental period of up to 80 days, which can mature after in vivo fat pad transplantation on a poly(lactide-co-glycolide) scaffold [74,83].

Recently, Tiwari et al. developed lung organoids from iPSCs in a time period of 60 days, by combining the protocols of Leibel et al. and Miller et al. [84]. Most notably, the authors described the presence of distal and proximal regions in the organoids, comprising basal and ciliated cells, as well as AT1 and AT2 cells. Early studies also demonstrated permissiveness of lung organoids to the VSV-based SARS-CoV-2 pseudovirus [84]. Furthermore, the use of different antiviral drugs, such as EK1, camostat and nafamostat, successfully inhibited the viral infection in lung organoids, demonstrating the suitability of this in vitro model to evaluate drug efficacy. The infection with SARS-CoV-2 led to a productive infection of the lung organoids, quantified via qRT-PCR. Moreover, several regulatory genes of the immune system were significantly upregulated upon SARS-CoV-2 infection, including STAT1 and 2, CCl5, CXCL10, IFNβ, IL-6 and IL-8 [84].

When primary cells are used instead of stem cells, the development of lung organoids can be completed in a shorter period of time and the organoids develop an adult phenotype. The embedding of primary cells in the basement membrane results in the 3D organization of human bronchial organoids within days. Notably, these organoids are suitable for passaging by mechanical disruption and re-embedding without significant alterations in the cellular composition. Sachs et al. described a consistent composition of basal, club, ciliated and goblet cells even in passage 19, with no significant change to passage 5, thus providing a clear advantage to the hPSC protocols [75]. However, this model has not yet been used to study SARS-CoV-2 infections.

Another study, by Suzuki et al., demonstrated the possibility of generating bronchial organoids from cryopreserved adult bronchial epithelial cells, thereby overcoming the need of lung biopsy samples [76]. Notably, only basal cells showed abundant expression of ACE2 and TMPRSS2, whereas ciliated cells did not express ACE2, resulting in SARS-CoV-2 infection of only basal cells. The productive infection was significantly reduced upon camostat treatment [76].

Many of the lung organoid models that are already in use for SARS-CoV-2 studies are based on development of the distal airway including AT2 cells, which show high ACE2 and TMPRSS2 expression, essential to study SARS-CoV-2 [77,78,79,80,81]. In the development of three-dimensional alveolosphere cultures or AT2 organoids, the apical, ACE2-containing side is located inside the 3D structure. Salahudeen et al. demonstrated the possibility of generating apical-out organoids, where reorganization of the organoids is introduced via an apical-out suspension culture polarization method [81]. The authors identified basal cells and club cells in their AT2 organoids, additional to AT2 cells by single-cell RNA sequencing. The alveolar organoids were established from primary lung parenchyma in several days and can be passaged every 3–4 weeks for 6 months. SARS-CoV-2 infection was reported in 10% of all infected apical-out organoids and only AT2 cells were permissive, with infectious progeny virus detected in organoid lysates and supernatants [81]. A similar approach was reported by Chen et al., who generated lung bud organoids by differentiation of hESCs into AT2 cells and few goblet cells, reporting the occurrence of extensive branching morphogenesis 2–3 weeks post Matrigel embedding (day 40+), but the absence of mature lung markers [82,85]. Furthermore, Han et al. used the protocol from Chen et al. amongst others to generate organoids and described the presence of ACE2, TMPRSS2 and FURIN. The lung organoids were successfully infected with SARS-CoV-2 virus encoding a luciferase reporter. Moreover, the lung organoids were treated with the antiviral drugs imatinib, MPA or QNHC, and subsequent infection with SARS-CoV-2 showed a dose-dependent virus entry inhibition [78].

Pei et al. developed human airway organoids and alveolar organoids from hESCs with abundant expression of ACE2 and TMPRSS2, thereby including the complete respiratory infection route of SARS-CoV-2. The authors reported productive SARS-CoV-2 infection of ciliated (90–95% of infected cells) and club cells (5–10% of infected cells) in the airway organoids, as well as AT2 cells (100% of infected cells) in alveolar organoids. The total number of infected cells in the airway organoids increased from 24 hours post infection (hpi) (ca. 26%) to 72 hpi (ca. 65%), whereas the percentage of infected AT2 cell remained stable at ca. 40%. The gene expression pattern of COVID-19 patients was recapitulated in the infected organoids, including downregulation of cell metabolism genes and upregulation of immune response genes. SARS-CoV-2 infection was successfully reduced upon treatment with remdesivir in a dose-dependent manner. Moreover, the neutralizing antibody CB6 was able to reduce viral replication. These results clearly indicate the suitability of the model to recapitulate SARS-CoV-2 infection and to analyze antiviral drug efficacy [77].

Katsura et al. developed a protocol that used purified AT2 cells from lung tissue, cultured in serum-free and feeder-free conditions as alveolospheres [79]. The medium is suitable to culture AT2 cells for more than 10 passages without alterations in morphology or self-renewal properties. The alveolar organoids were infected with SARS-CoV-2-GFP and released infectious virions with a peak after 24 h. In total, around 30% of the alveolospheres were successfully infected with SARS-CoV-2, with infection leading to a downregulation of SFTPC compared to controls. Significant cell death was also observed in infected and surrounding cells. Transcriptome analysis of infected organoids revealed the enrichment of multiple IFN-I and III transcripts, STAT1 and STAT2, chemokines and apoptosis-related genes. On the other hand, no upregulation of IFN-II transcripts was detected. Moreover, DNA replication- and cell-cycle-related genes were downregulated. Remarkably, the authors described similar IFN expression patterns in AT2 cells from infected organoids and human lungs. When the alveolar organoids were treated with IFNs before infection, viral replication was significantly reduced, and the contrary was detected upon pre-treatment with the IFN signaling inhibitor ruxolitinib. Lastly, the authors suggest the co-culture of alveolospheres with immune cells to study their interactions upon viral infection [79].

A similar alveolosphere protocol was developed by Youk et al., enabling the growth of human AT2 cells in three-dimensional culture after embedding in Matrigel [80]. For SARS-CoV-2 infection, the organoids were disrupted to enable apical surface accessibility. Infection led to the release of viral particles within 24 hpi, with preserved cell viability. Quantification revealed that 94% of all organoids and 61% of all cells were infected. The infection influenced gene expression, especially 3 dpi, with significant upregulation of ISGs activated by type I and III interferons, which is in accordance with data published by Katsura et al. [79,80].

To increase the viability and maturation of the organoids, they can be engrafted in vivo in immunodeficient mice, as the vascularization of the host invades the incorporated tissue, previously demonstrated for lung organoids [74,83], brain organoids [86], and kidney organoids [87]. Alternatively, engineered organoid vascularization can significantly increase oxygen and nutrient supply and can enhance growth, as it is already performed for kidney organoids [88].

To conclude, the development of the organoids is cost- and-time intensive but serves as an authentic and highly functional in vitro model to study SARS-CoV-2. Nonetheless, the high organoid variability in size and cellular composition must be considered.

### 2.4. Lung-on-a-Chip—The Future?

**Figure 4:**
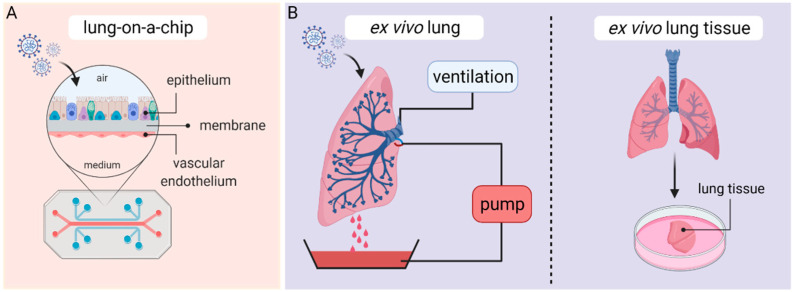
Lung-on-a-chip (**A**) and ex vivo (**B**) culture techniques. Figure was created with BioRender.com (accessed on 26 March 2021).

Microengineered organ-on-a-chip technology provides a cell culture system in a microfluidic setup, enabling the recapitulation of cellular interactions in a vascularized environment, closely mimicking organ function. Organ-on-a-chip models are potentially able to create a platform more suitable for drug screenings and to translate results to humans. The biomimetic lung-on-a-chip system enables co-culturing of epithelial and endothelial cells in a biointerface, allowing crosstalk between both layers (Figure 4A). Within the chip, the microvascular endothelial cell layer is located in a vascular channel, whereas the alveolar epithelial layer is cultured in a second channel on an air–liquid interface. Both channels are separated by a porous polydimethylsiloxane membrane, coated with an extracellular matrix [89,90].

This model is suitable to study immune responses and disease pathology in a physiologically relevant environment. Recently, organs-on-chips have been gaining importance, as their impact for virology has been extensively discussed [91]. In the past, the lung-on-a-chip technique was used to study influenza virus infections and was useful to study not only virus–host interactions, but the recruitment of host immune cells and host-immune responses upon infection [92]. These results pave the way for further in vitro studies, including SARS-CoV-2 research [93].

The first studies using lung-on-chip technology to study SARS-CoV-2 have already been performed which addressed lung microvasculature as key target in COVID-19 pathogenesis [94]. Thacker et al. used a vascularized lung-on-a-chip model composed of alveolar epithelial cells and microvascular endothelial cells and revealed a significant increase of ACE2 and Neuropilin-1 (NRP1) expression in these cells upon culturing on a chip. SARS-CoV-2 infection with a low MOI led to downregulation of ACE2 in the epithelial compartment and NRP1 in both the endothelial and epithelial compartment, whereas TMPRSS2 expression was upregulated. Moreover, a small number of infectious viruses were released apically, and no release occurred to the vascular effluent. In contrast, the viral copy numbers detected intracellularly were significantly increased in both endothelial and epithelial compartment. Ultimately, the authors suggested a rapid transfer of viral particles from the epithelial site of infection to the endothelial layer by basolateral transfer [94]. With progression of the time post infection, alterations in the endothelial layer increased. For example, reduced cell–cell contacts and loss of cell confluency were reported 2–3 dpi. Several pro-inflammatory responses that are related to NF-κB are elevated, whereas antiviral IFN responses were suppressed. Interestingly, constant IL-6 secretion was detected in the vascular effluent, even upon co-culturing the epithelial cells with CD14^+^ macrophages. The authors report an infection pattern which agrees with the reported clinical disease [94].

In another study, Zhang et al. used the lung-on-chip technology to study SARS-CoV-2 infection with a high MOI, revealing high viral infection in epithelial cells but not in endothelial cells [95]. The authors reported IFN-I immune response in epithelial cells and JAK-STAT signaling in endothelial cells upon infection. Elevated levels of IL-6 and IL-8 were detected in the vascular channel. After addition of PBMCs to the vascular channel, infection with SARS-CoV-2 revealed the attachment of CD14^+^ monocytes to the endothelial compartment. Most significantly, increased levels of IL-6, IL-1β and TNFα were detected upon PBMC presence [95].

Both studies show the suitability of the lung-on-a-chip model to study SARS-CoV-2 infection, as the model can recapitulate the alveolar capillary barrier function and injury, SARS-CoV-2 infection and inflammatory response, recruitment of immune cells and cross-talk among the epithelium–endothelium barrier [94,95]. However, among these advantages, the costly generation of lung-on-a-chip models limits large-scale applications of this technique. Most importantly, lung-on-a-chip models have yet to be established as an easy-to-use cell culture tool in a standard research setting.

## 3. Ex Vivo Lung Perfusion (EVLP) Models and Human Lung Tissue Explants

In recent years, human ex vivo lung perfusion (EVLP) models and human lung tissue explants have become valuable preclinical platforms to carry out studies on human respiratory diseases. EVLP models were initially developed in the 1950s and used to evaluate, preserve and recondition donor lungs before transplantation [96]. EVLP lungs are usually obtained from cardiac death donors or brain-dead transplant donors after the organ has been rejected for transplantation and are maintained at normothermic physiological conditions by ex vivo perfusion and ventilation according to the Lund protocol [97]. In brief, human donor lungs are preserved at 37 °C and perfused by connecting a peristaltic pump to the pulmonary artery (Figure 4B). Since perfusate solution drains passively from the pulmonary veins, a reservoir is placed at the bottom of the perfusion chamber and perfusate is recovered and recycled for perfusion flow. In addition, the primary bronchus gets cannulated with an endotracheal tube to inflate the lung with room air or a mix of 95% O_2_ and 5% CO_2_ via continuous positive airway pressure (CPAP) or positive-pressure ventilation [98,99].

Despite the fact that clinical applications of EVLP systems were only established in the early 2010s, ex vivo lung perfusion models have since advanced into a suitable model to study mechanisms of acute lung injury, bacterial pneumonia and bacteremia of Gram-positive and Gram-negative organisms [98,100,101], tolerability and delivery of new pulmonary therapeutics, as well as cell therapeutic interventions [100,102,103,104]. Still, integrating and installing EVLP platforms into BSL-2 and BSL-3 facilities required to safely study viral infections has not been easy and only a few attempts have been made so far. For example, a preliminary study by Chen et al. examined the therapeutic efficacy of Nanotrap-antibodies in inhibiting SARS-CoV-2 infection using an EVLP system. By injecting either with SARS-CoV-2 spike pseudotyped lentivirus carrying a luciferase reporter gene or lentivirus incubated with Nanotrap-antibodies into different lobes of a healthy, non-transplantable human donor lung and subsequent 8 h perfusion, they were able to demonstrate SARS-CoV-2 pseudovirus inhibition by Nanotrap-antibodies. Of note, the same study was able to further verify their results in traditional cell culture and animal models [105].

As an alternative to EVLP systems, lung explants can be used to study SARS-CoV-2 infections. Lung explants are usually obtained from human lung transplants dissected into smaller pieces or from patients who underwent surgical operations. These tissue slices are then directly used for infection experiments, making them easier to handle in a biosafety laboratory setting, unlike EVLP systems.

In recent years, lung explants have been used to study viral replication, cellular tropism, immune responses and new therapeutic approaches for various viruses, including influenza A viruses [106,107,108,109,110,111,112], adenoviruses [113,114] and coronaviruses [115,116,117]. For instance, human lung tissue explants have been used to study viral replication and cellular tropism of highly pathogenic MERS-CoV. Hocke et al. [115] and Chan et al. [116] showed MERS-CoV replication in bronchial epithelial cells, AT1 cells, AT2 cells, and endothelial cells in lung tissues infected with human or dromedary virus isolates. In addition, Hocke et al. found structural lung damage caused in apoptotic AT2 cells by MERS-CoV infection. Notably, comparison of ex vivo study results and autopsy reports confirmed the results regarding viral tropism and lung damage [117].

Since the emergence of SARS-CoV-2, numerous studies have also been conducted investigating virus replication [118] and antiviral effects of small molecule inhibitors [119] in lung tissue explants. For instance, Chu et al. used dissected lung organs and challenged them with SARS-CoV and SARS-CoV-2 isolates [40]. Both viruses were able to replicate in the lung tissue and virus titers increased steadily from 2 h to 48 h; however, a 3.2-fold higher titer of SARS-CoV-2 virus particles compared to SARS-CoV was observed. AT1 and AT2, as well as alveolar macrophages, were revealed as the main viral targets for both viruses.

While ex vivo platforms and tissue explants authentically portray the pathophysiology of lung diseases, these approaches are also associated with a number of drawbacks (Table 2). Even though only 15–25% of the total lung donor pool meet the criteria for transplantation [120,121]—i.e., an estimated 75–85% would theoretically be available for EVLPs—this does not mitigate the fact that the number of suitable lungs for EVLP is still fairly small as a result of geographical, financial and ethical limitations. Additionally, reproducibility is constrained due to the significant heterogeneity in human lung tissue (e.g., sex, age, smoking history, underlying diseases of donor) and pre-procurement variables (e.g., trauma, ventilator-induced lung injury and ventilator-associated pneumonia). Moreover, current perfusion protocols are restricted by short perfusion times of up to 12 h. Nevertheless, ex vivo platforms have demonstrated to be especially useful to study mechanisms, pathways and targets identified through in vitro systems and early animal models before having to commit to the complexity, time, and costs of human trials [98,122], and, most importantly, address an unmet need in the field of translational research [122].

## 4. Conclusions

In this review, we summarized the current state of the art in vitro models to study respiratory diseases and discuss benefits and drawbacks which need to be considered when studying viral infections, with a focus on SARS-CoV-2. Of note, we have not covered in vivo animal studies. It is worth mentioning that the presented in vitro and ex vivo models are not substituting, but are rather complementing each other when studying respiratory viruses. Cell culture models have been and will remain to be invaluable tools for studying virus infection and they help us to solve pressing questions about virus pathology, viral replication, disease mechanisms and drug efficiency.

## Figures and Tables

**Table 1:** Overview of cell lines most commonly used to study SARS-CoV-2.

Cell Lines	Origin	Characteristics	ACE2 Expression	Reference
Vero E6	Kidney epithelial cells extracted from African green monkey (*Chlorocebus* sp.)	Interferon-deficient (do not secrete IFNα or IFNβ when infected by viruses), non-tumorigenic, pseudodiploid karyotypes	+++	[36,37]
Calu-3	Human lung adenocarcinoma	Epithelial cells	+	[36]
A549	Human lung adenocarcinoma	Epithelium-like, hypotriproid, synthesizes comparably large amounts of lecithin	−	[37,38]
Caco-2	Human colorectal adenocarcinoma	Epithelium-like, upon reaching confluence, the cells express characteristics of enterocytic differentiation; express heat stable enterotoxin and epidermal growth factor	++	[36]
HEK-293T	Human embryonic kidney	Epithelial cells, highly transfectable, contains the SV40 T-antigen, widely used for retroviral production, gene expression and protein production	−	[39]
Huh-7	Human hepatocellular carcinoma	Epithelial cells, tumorigenic	+	[37]

The symbols indicate high (+++), moderate (++), mild (+) and low (−) applicability.

**Table 2:** Summary of advantages and disadvantages of the elucidated lung models in this review.

	Continuous Cell Lines	hAEC-ALI Cultures	Lung Organoids	Lung-on-Chip	EVLP/Lung Tissue Explants
Availability	+++	++	++	+	+
Affordability	+++	++	+	+	−
Authenticity/Physiological relevance	+	+++	++	+++	+++
Handling	+++	++	++	+	+
Reproducibility	+++	++	+	+	−
Genetic manipulation	+++	+	++	+	−
Application	Virus propagation, diagnostics, high-throughput drug screenings, host–pathogen interactions	Cell tropism, disease pathology immune response, transcriptomics, cell signaling, virus–host interactions, drug testing	Cell tropism, disease pathology, transcriptomics, cell signaling, virus–host interactions, drug testing	Immune and inflammatory responses, disease pathology, virus–host interactions, cross-talk, drug testing	Drug testing, host–pathogen interactions, cellular tropism

The symbols indicate high (+++), moderate (++), mild (+) and low (−) applicability.

## Data Availability

Not applicable.
